# Heredity

**Published:** 1899-02

**Authors:** 


### Heredity.

Dr. A. L. Benedict, in the *Medical Times* for July, 1898, in an article on heredity, says many of the so-called nervous and mental diseases show genuine marks of heredity, and it is significant that impressionability and general loss of nervous control are inherited rather than definite diseased conditions. Genuine anatomic anomalies are also hereditary in much the same way as physiologic defects of the nervous system. In these cases the hereditary taint usually affects only one or two members of a generation. If the family is afflicted generally, a few generations usually suffice to extinguish the trait, either with the family or by the superior strength of normal tendencies. In some cases hereditary taint, such as insanity, prevents the marriage of most of those actually afflicted and of the women of a family, while the unaffected males marry, bringing in healthy blood. By the law of crossed heredity, the neurotic taint would tend to be transmitted to female offspring, while the males would inherit largely from wholesome maternal stock. Thus, theoretically, the surname line would tend toward purification.

In summing up, the writer would emphasize the following beliefs:

1. Much of what is commonly ascribed to heredity should properly be credited to infection, environment, or even chance.

2. True heredity deals with general and acquired traits rather than with disease which is essentially foreign to the organism.

3. On account of the vast number of ancestors involved, the introduction of fresh blood by intermarriage, and the crossing of hereditary tendencies from one sex to another, there is, on the whole, a tendency to reversion to general characteristics and to purification from taints.

4. A disease to be hereditary must depend on some intrinsic physiologic or anatomic abnormality, and not essentially on infection. If it manifests itself before the period of reproduction, the tendency must disappear either with the destruction of the family or by the superior force of the normal tendencies. If it does not interfere with reproduction, it is still amenable to hygienic precautions.

5. The only intelligent knowledge of hereditary must come from a close study of genealogy, carried on impartially and without the present unworthy incentives.—*Med. Age.*

